# Is the pay-for-performance program associated with better quality of life among type 2 diabetes patients, including those with gastrointestinal conditions, in Taiwan? A cross-sectional survey

**DOI:** 10.1371/journal.pone.0328910

**Published:** 2025-08-22

**Authors:** Shao-Hua Kuang, Tsung-Tai Chen, Wen-Ya Ma, Szu-Tah Chen, Meng-Han Shih, Ching-Chieh Su, Wei-Chih Su, Li Ying Huang, Ya-Seng (Arthur) Hsueh, Vinchi Wang

**Affiliations:** 1 Superintendent Office, Cardinal Tien Hospital, New Taipei City, Taiwan; 2 Department of Public Health, College of Medicine, Fu Jen Catholic University, New Taipei City, Taiwan; 3 Division of Endocrinology, Department of Internal Medicine Cardinal Tien Hospital, New Taipei City, Taiwan; 4 Division of Endocrinology and Metabolism, Department of Internal Medicine, Chang Gung Memorial Hospital at Linkou, Taoyuan, Taiwan; 5 College of Medicine, Chang Gung University, Taoyuan, Taiwan; 6 Dr. Su Diabetes Clinic, New Taipei City, Taiwan; 7 Department of Gastroenterology, Taipei Tzu-Chi Hospital, New Taipei City, Taiwan; 8 Division of Endocrinology and Metabolism, Department of Internal Medicine, Fu Jen Catholic University Hospital, Fu Jen Catholic University, New Taipei City, Taiwan; 9 School of Medicine, College of Medicine, Fu Jen Catholic University, New Taipei City, Taiwan; 10 Centre for Health Policy, Melbourne School of Population and Global Health, The University of Melbourne, Melbourne, Victoria, Australia; 11 Department of Neurology, Cardinal Tien Hospital, New Taipei City, Taiwan; University of Lisbon, Institute of Social and Political Sciences, PORTUGAL

## Abstract

**Background:**

Many studies have shown that Taiwan’s diabetes pay-for-performance (P4P) program can improve several patients’ clinical outcomes; however, it remains unclear whether P4P initiatives yield effects on health-related quality of life (HRQoL) for all diabetes patients as well as those with comorbidities, especially comorbid gastrointestinal conditions.

**Purpose:**

In this study, we evaluated the effects of the diabetes P4P program on QoL among all patients as well as the effects of this program on QoL among patients with comorbid gastrointestinal conditions.

**Methods:**

Data were collected across 6 hospitals. We used the five-level version of the EuroQol-5 Dimension (EQ-5D-5L) to obtain scores for health disutility. The scores for these measures were subsequently predicted via a generalized linear model (GLM) with a gamma distribution. We also applied propensity score weighting (PSW) to adjust for potential selection bias.

**Results:**

After PSW, P4P program participation was found to be significantly correlated with lower EQ-5D disutility scores among all patients (parameter = −0.169, [−0.325, −0.012], *P* = 0.034). Additionally, P4P program participation was significantly correlated with a lower EQ-5D disutility score among patients with comorbid gastrointestinal conditions (parameter = −0.604, [−0.891, −0.317], *P* < 0.001).

**Conclusion:**

Our findings suggest that participation in the diabetes P4P program is associated with improved HRQoL among all patients with type 2 diabetes, including those with gastrointestinal comorbidities. A P4P model incorporating continuing education, coordinated care, and regular follow-up may be particularly beneficial in enhancing HRQoL for patients with gastrointestinal complications.

## Introduction

The prevalence of diabetes in Taiwan was approximately 13.1% in 2021 [1]. Patients with diabetes are more likely to have comorbidities, such as gastrointestinal conditions [2]. For example, patients with diabetes had a significantly higher prevalence of hepatitis C infection than hepatitis B infection [3]. The prevalence rate of hepatitis C infection among patients with diabetes was 6.1% in Taiwan, which is higher than the global prevalence rate of 4.2% [4]. Moreover, patients with diabetes and hepatitis C infections may have a greater risk of developing cirrhosis [5], which can lead to the development of hepatocellular carcinoma [6], increased mortality [4], and comorbidities that negatively impact patients’ quality of life (QoL) [7,8]. For example, diabetes patients with hepatitis B may exhibit poor glycemic control and poor QoL [9]. Regarding QoL, during this era of patient-centered care, the National Institute of Diabetes and Digestive and Kidney Diseases (NIDDK) and the American Diabetes Association (ADA) are currently highlighting the importance of patient-reported outcome (PRO) research, including QoL research, among patients with type 2 diabetes [10]. QoL is a multidimensional, subjective and dynamic measure that is used to evaluate what patients want from life, understand their perceived health status and assess how the disease affects their lives [11]. Furthermore, QoL measures can be used to detect individual psychosocial problems that may impact a patient’s therapeutic response [12]. Therefore, understanding whether interventions can improve QoL is important.

Many studies have shown that Taiwan’s diabetes pay-for-performance (P4P) program can improve common patients’ different clinical outcomes [13–17], and P4P in diabetes management has been shown to be cost-effective [16,17,18]. However, these studies have not shown that participants in P4P programs experience significantly better quality of life. Some studies have explored the benefits of P4P programs for patients with diabetes and multiple chronic conditions, but they have not specifically examined the outcomes for diabetic patients with comorbid gastrointestinal conditions [19,20]. Therefore, there is a lack of clarity regarding whether P4P initiatives affect the QoL of patients with comorbidities, especially those with gastrointestinal comorbidities. In this study, we evaluated the effect of the diabetes P4P program on QoL among all type 2 diabetes patients as well as the effects of this program on QoL among patients with gastrointestinal comorbidities.

## Methods

### Study design

In this cross-sectional survey study, data were collected from patient surveys and record reviews across 6 hospitals, including two tertiary hospitals and four regional hospitals in northern Taiwan, from October to December 2016.

### Setting

The National Health Insurance Administration (NHIA) integrated external incentives with a shared care network (SCN) to form the diabetes P4P program in 2001, which not only enforces the execution of suggested activities by the SCN but also suggests adherence to guidelines for physicians conducting the necessary examinations. The incentives (US $108) include three follow-up fees (total US $21), a one-time yearly evaluation fee (US $27), and physician fees that are paid four times, once for every patient visit except for visits to stand-alone clinics (total US $60) [21]. Physicians have the autonomy to decide whether to enroll treated patients with diabetes in the P4P program.

Additionally, the diabetes P4P program in Taiwan has many distinctive features of the chronic care model (CCM), which could result in better patient outcomes [21], such as coordination between diabetes-related professionals, autonomy, and the assembly of family resources. The diabetes P4P program in Taiwan has embedded this CCM-based disease management program (DMP) to lead to better patient outcomes than the hepatitis P4P program without an embedded DMP [21,22]. The P4P program-enrolled patients can be treated by teams consisting of trained professionals, including providers (specialists in endocrinology, family medicine or internal medicine), health educators, dieticians, and laboratory technicians. The teams motivate and support patients’ autonomy by encouraging healthy behavior depending on the patient’s lifestyle and preferences [21,23]. Detailed descriptions of the diabetes P4P program in Taiwan can be found in a previous article [21].

### Participants

The inclusion criteria were as follows: (1) had a diagnosis of diabetes (ICD-9-CM 250); (2) were 18 years or older; and (3) had sufficient mental capacity to respond to the complete questionnaire. In this study, 1512 questionnaires were distributed after informed consent was obtained, and 1512 valid questionnaires were returned. The final analytical sample consisted of 1512 individuals. We excluded 30 subjects because of missing information regarding income and the EQ-5D index. Therefore, the final sample included 1,482 individuals. We further stratified these patients based on whether they participated in a P4P program during the year of enrollment in this study. Patients in the P4P group were also required to be enrolled for at least 1 year after the first enrollment day. The remaining patients not included in the P4P group were categorized into the control group (non-P4P group).

### Variables and data sources/measurement

We accounted for common patient demographic characteristics and diabetes-related variables. The former demographic variables include age, sex, income, employment, marital status, education, and urbanization of the residence area. The communities in Taiwan are stratified into seven urbanization categories according to the Taiwanese National Health Research Institute (NHRI). Cluster analysis was used to divide these areas into urbanization groups from the highest level of urbanization to the lowest level as follows: high-level, median-level, emerging, common, aging, agricultural, and remote areas [24]. The latter diabetes-related variables included treatment, multiple shots of insulin per day (yes or no), any complications [25], and previous hospital admission within one year. The treatments included 4 categories: exercise and diet control, oral medications only, oral medications and use of insulin, and use of insulin only.

We assessed gastrointestinal conditions by using the chronic illness with complexity (CIC) index, which assesses peptic ulcers, inflammatory bowel disease, diverticulitis, chronic hepatitis, gallbladder disease and gallstones [2]. Additionally, we used the EQ-5D 5 L to obtain health utility scores; the EQ-5D 5 L is suitable for utility measurements for diabetes patients [26]. The scores of all 5 dimensions of the EQ-5D index were combined with preference weights derived from the Taiwanese value sets [27].

The common patient demographic characteristics, patient complexity (gastrointestinal conditions and complications) and EQ-5D 5 L scores were derived mainly from the patients’ questionnaires. To ensure consistency among the interviewers in this study, we have interviewer training sessions, which were held prior to the commencement of the interviews. During these sessions, each question and its corresponding response options were read aloud. We trained the interviewers in important details of the EQ-5D questionnaire, such as the rationale behind it and how the EQ-5D questionnaire should be administered. Patient complexity was also clarified and explained in the training sessions. To obtain complete information about gastrointestinal conditions for patients with diabetes, we provided a list of the diseases described above to allow patients to select whether they had at least one of the gastrointestinal conditions, and we asked patients to verify that the reported diseases or conditions had ever been diagnosed by physicians. After the completion of the questionnaire, the interviewers verified the gastrointestinal conditions that the patients provided by asking patients the same questions again. If the respondents could not read or were unfamiliar with the questionnaire, we asked the interviewers to read the entire questionnaire in a manner that the interviewees could understand. After completing the questionnaire, we extracted each patient’s P4P status from the medical records.

### Statistical methods

The variance inflation factor (VIF) was calculated to determined whether there was multicollinearity between independent variables [28]. Regarding the skewness and ceiling effect of the EQ-5D index, in general, the utility values are expected to be skewed, as many patients report perfect health [29,30]. To address these problems, we first conducted a Kolmogorov–Smirnov test to examine normality. Then, we used a two-part model, which was estimated using the STATA *twopm* command, to adjust the skewness and ceiling effects of the EQ-5D index. In general, the two parts of the model were 1) a logistic regression and 2) a generalized linear model (GLM) using backward elimination. The outcome was the disutility score. The logistic regression modeled the probability of disutility (1-EQ-5D index) for transformation of the probability of disutility because too many respondents reported a perfect EQ-5D index (Pr (y > 0 | x)). The second part of the model utilized a GLM with a gamma distribution and a log link function under the condition of probability of disutility [22,29,31]. The independent variables included common patient characteristics and diabetes-related variables, as described in the variables section.

To prevent selection bias in the P4P group, we applied propensity score weighting (PSW) with inverse probability weighting (IPW) to appropriately weight patients in the P4P intervention and control groups [32], and the resulting propensity score was used as a control variable in the two-part model. This research used the aforementioned characteristics of patients to generate inverse probability weighting (propensity score) for each patient via logistic regression. Compared with the matching method, weighting the propensity score has the advantage of incorporating most of the analytical observations and can increase precision in estimating treatment effects [33]. IPW involves weighting by the inverse probability of receiving the study intervention (1/propensity score for the P4P group and 1/(1 − propensity score) for the non-P4P group) [32]. All analyses were conducted via Stata Statistical Software version 15. The study was approved by the Institutional Review Board (TCHIRB-104–12123-E) of the Taipei City Hospital.

## Results

Table 1 shows that the average age of the patients was 63 years. 45% of the patients were P4P program patients; approximately 54% were male; 64% had an income less than US$1,000 per month; 28% had a full-time job; 77% were married; 62% were religious; 53% had an education level below junior high school; 69% lived in an area with high-level urbanization and took oral medication only(72%) without the use of insulin; 10% took multiple shots of insulin per day; 19% had gastrointestinal conditions; 30% had any complications; and 4% had previous hospital admissions within one year due to diabetes. Additionally, there were significant differences in employment, marital status, level of urbanization, and treatment method for diabetes between P4P patients and non-P4P program patients. The patients in the P4P group had a significantly greater average EQ-5D index (0.935) than did the patients in the control group, who had an average EQ-5D index of 0.90 (*P* < 0.001) (data not shown).

**Table 1:** Characteristics of the study participants.

	Total (n = 1482)	P4P (n = 674)	Non-P4P (n = 808)	*P* value
Age, y (SE*)	62.6(0.4)	64.4(0.4)	61.1(0.4)	<0.001
Male	799(53.9)	380(56.4)	419(51.9)	0.08
Income per month				
< U.S.$1,000	942(63.6)	442(65.6)	500(61.9)	0.49
U.S.$1,000-$2,000	397(26.8)	171(25.4)	226(28.0)	
U.S.$2,001-$3,333	104(7.0)	43(6.4)	61(7.6)	
> U.S.$3,333	39(2.6)	18(2.8)	21(2.6)	
Employment				
Full-time without a shift	349(23.6)	153(22.7)	196(24.3)	0.02
Full-time with a shift	60(4.1)	20(3.0)	40(5.0)	
Retired	522(35.2)	266(39.5)	256(31.7)	
Part-time	53(3.6)	20(3.0)	33(4.1)	
Self-employed	122(8.2)	55(8.2)	67(8.3)	
Others (e.g., loss of job or housekeeper)	376(25.4)	160(23.7)	216(26.7)	
Marital status				
Married	1135(76.6)	536(79.5)	599(74.1)	0.03
Unmarried	136(9.2)	48(7.1)	88(11.0)	
Widowed	135(9.1)	61(9.1)	74(9.2)	
Other (e.g., separated, divorced)	76(5.1)	29(4.3)	47(5.8)	
Education				
Below elementary school^a^	488(33.0)	231(34.3)	257(31.8)	0.17
Junior high school	297(20.0)	143(21.2)	154(19.1)	
Junior college	138(9.3)	54(8.0)	84(10.4)	
Senior high school^b^	378(25.5)	174(25.8)	204(25.3)	
University	145(9.8)	62(9.2)	83(10.3)	
Graduate or doctoral degree	36(2.4)	10(1.5)	26(3.2)	
Religion	921(62.1)	435(64.5)	486(60.1)	0.08
Level of urbanization				
High-level	1026(69.2)	519(77.0)	507(62.8)	<0.001
Median-level	290(19.6)	96(14.2)	194(24.0)	
Emerging	129(8.7)	48(7.1)	81(10.0)	
Common	37(2.5)	11(1.6)	26(3.2)	
Treatments				
Exercise and diet control	83(5.6)	16(2.4)	67(8.3)	<0.001
Oral medications only	1071(72.3)	525(77.9)	546(67.6)	
Oral medications and use of insulin	265(17.9)	107(15.9)	158(19.6)	
Use of insulin only	63(4.3)	26(3.9)	37(4.6)	
Multiple shots of insulin per day	144(9.7)	57(8.5)	87(10.8)	0.13
Gastrointestinal conditions	284(19.2)	128(19.0)	156(19.3)	0.88
No complications	1034(69.8)	467(69.3)	567(70.2)	0.71
Previous hospital admission within one year	54(3.6)	15(2.2)	39(4.8)	0.008

Note: Below elementary school^a^ includes people who cannot read; senior high school^b^ includes vocational senior high schools.

As shown in S1 Table of the supporting information, after applying PSW, the absolute values of the standardized mean differences (SMDs) between the P4P and non-P4P groups significantly decreased for every variable and its related categories. The largest of these differences is 0.022 in absolute value, which is much lower than the upper limit of 0.25 recommended by another study.

Table 2 shows the distribution of EQ-5D-5L responses of diabetes patients and compares the results on the basis of the status of P4P program participation. Compared with non-P4P group patients, patients in the P4P group presented higher percentages of level 1 in all five domains. The differences between the two groups were statistically significant in two domains: usual activity (*P* = 0.03) and anxiety/depression (*P* = 0.001). The distribution of EQ-5D-5L responses among diabetes patients and a comparison of the results between patients with and without gastrointestinal comorbidities are shown in Table 3. Compared with patients without gastrointestinal comorbidities, patients with gastrointestinal comorbidities presented lower percentages of level 1 in all five domains. The differences between the two groups were statistically significant in all domains.

**Table 2:** Distribution of EQ-5D-5L responses by P4P participation groups.

	P4P (%)	Non-P4P (%)	*P* value^a^
	(n = 674)	(n = 808)	
Mobility			
Level 1	630(93.5)	727(90.0)	0.11
Level 2	33(4.9)	56(6.9)	
Level 3	8(1.2)	21(2.6)	
Level 4	3(0.5)	3(0.4)	
Level 5	1(0.1)	1(0.1)	
Self-care			
Level 1	662(98.2)	779(96.4)	0.06
Level 2	7(1.0)	17(2.1)	
Level 3	2(0.3)	9(1.1)	
Level 4	3(0.5)	1(0.1)	
Level 5	0(0)	2(0.3)	
Usual activity			
Level 1	651(96.6)	755(93.4)	0.03
Level 2	15(2.2)	36(4.5)	
Level 3	4(0.6)	11(1.4)	
Level 4	4(0.6)	3(0.4)	
Level 5	0(0)	3(0.4)	
Pain/discomfort			
Level 1	451(66.9)	496(61.4)	0.08
Level 2	198(29.4)	260(32.2)	
Level 3	23(3.4)	48(5.9)	
Level 4	1(0.2)	3(0.4)	
Level 5	1(0.2)	1(0.1)	
Anxiety/depression			
Level 1	574(85.2)	637(78.8)	0.001
Level 2	92(13.7)	150(18.6)	
Level 3	4(0.6)	16(2.0)	
Level 4	4(0.6)	1(0.1)	
Level 5	0(0)	4(0.5)	

Note: *P* value.

^a^: *P* values were derived from the chi-square test.

**Table 3:** Distribution of EQ-5D-5L responses in patients with diabetes with/without gastrointestinal comorbidities.

	Gastrointestinal (%)	Without gastrointestinal (%)	*P* value^a^
	(n = 284)	(n = 1198)	
Mobility			
Level 1	254(89.4)	1103(92.1)	0.026
Level 2	17(6.0)	72(6.0)	
Level 3	11(3.9)	18(1.5)	
Level 4	1(0.4)	5(0.4)	
Level 5	1(0.4)	0(0)	
Self-care			
Level 1	270(95.1)	1171(97.7)	<0.001
Level 2	4(1.4)	20(1.7)	
Level 3	7(2.5)	4(0.3)	
Level 4	1(0.4)	3(0.3)	
Level 5	2(0.7)	0(0)	
Usual activity			
Level 1	265(93.3)	1141(95.2)	0.021
Level 2	8(2.8)	43(3.6)	
Level 3	6(2.1)	9(0.8)	
Level 4	3(1.1)	4(0.3)	
Level 5	2(0.7)	1(0.1)	
Pain/discomfort			
Level 1	153(53.9)	794(66.3)	0.002
Level 2	110(38.7)	348(29.0)	
Level 3	19(6.7)	52(4.3)	
Level 4	1(0.4)	3(0.3)	
Level 5	1(0.4)	1(0.1)	
Anxiety/depression			
Level 1	215(75.7)	996(83.1)	0.006
Level 2	60(21.1)	182(15.2)	
Level 3	4(1.4)	16(1.3)	
Level 4	3(1.1)	2(0.2)	
Level 5	2(0.7)	2(0.2)	

Note: *P* value.

^a^: *P* values were derived from the chi-square test.

Table 4 shows the results of the analysis of the disutility EQ-5D index via a two-part model among all patients and those with gastrointestinal comorbidities after PSW adjustment. P4P program participation was significantly correlated with lower EQ-5D disutility scores among all patients (parameter = −0.169, [−0.325, −0.012], *P* = 0.034). With respect to patient demographic variables, patients with higher education had lower disutility scores for all patients (university, parameter = −0.202, [−0.378, −0.025], *P* = 0.025; reference: below elementary school). Patients with jobs had lower disutility scores in the total cohort (full-time without shift, parameter = −0.396, [−0.548, −0.244], *P* < 0.001; full-time with shift, parameter = −0.399, [−0.700, −0.098], *P* = 0.009; self-employed, parameter = −0.296, [−0.533, −0.060], *P* = 0.014; reference: others [loss of job or housekeeper]). With respect to diabetes-related variables, patients’ treatment with exercise and diet control had lower disutility scores (parameter = −0.310, [−0.563, −0.058], *P* = 0.016; reference: oral medications only).

**Table 4:** Estimated coefficients for the disutility EQ-5D index for all patients and those with gastrointestinal comorbidities.

	Total patients (n = 1,482)		With gastrointestinal comorbidities (n = 284)	
	Parameter (95% CI^c^)	*P* value	Parameter (95% CI^c^)	*P* value
**P4P** ^a^	−0.169 (−0.325, −0.012)	0.034	−0.604 (−0.891, −0.317)	<0.001
**Patient demographic variables**				
Income (Ref: < U.S.$1,000)				
Income U.S.$2,001-$3,333			−0.777 (−1.055, −0.498)	<0.001
Education (Ref: Below elementary school)				
University	−0.202 (−0.378, −0.025)	0.025		
Employment (Ref: Others^b^)				
Full-time without shift	−0.396 (−0.548, −0.244)	<0.001		
Full-time with shift	−0.399 (−0.700, −0.098)	0.009		
** **Self-employed	−0.296 (−0.533, −0.060)	0.014		
**Diabetes-related variables**				
** **Treatment (Ref: Oral medications only)				
** **Exercise and diet control	−0.310 (−0.563, −0.058)	0.016		
Multiple shots of insulin per day (Ref: No or single shot of insulin)			0.810 (0.256,1.364)	0.004

Note: P4P

^a^:pay-for-performance; Employment (Ref: Others

^b^):loss of job or housekeeper; CI

^c^:confidence interval

Similarly, P4P program participation was also significantly correlated with lower EQ-5D disutility scores among patients with gastrointestinal comorbidities (parameter = −0.604, [−0.891, −0.317], *P* < 0.001). With respect to patient demographic variables, higher-income patients had lower EQ-5D disutility scores in the gastrointestinal comorbidity cohort (income U.S.$2,001-$3,333, parameter = −0.777, [−1.055, −0.498], *P* < 0.001; reference: income < U.S.$1,000). With respect to diabetes-related variables, patients with multiple shots of insulin per day had higher disutility scores (parameter = 0.810, [0.256, 1.364], *P* = 0.00; reference: no or a single shot of insulin).

## Discussion

Previous studies have demonstrated that diabetes P4P programs could be associated with better clinical outcomes. However, no studies have proven that diabetes P4P programs could also be associated with better QoL among patients with gastrointestinal comorbidities. We not only detected positive effects of the diabetes P4P program on QoL among all patients with diabetes but also observed significant positive effects of the P4P program on QoL among patients with gastrointestinal comorbidities.

Previous studies on the quality of life (QoL) of patients with diabetes have shown that factors such as employment [34], higher income [35], and higher educational attainment [36] were positively associated with EQ-5D index values. Consistent with these findings, we observed similar patterns in our analysis. In addition, patients who managed diabetes through exercise and diet control, rather than oral medications, and those receiving only a single daily insulin injection, as opposed to multiple injections, reported better QoL. These differences likely reflect variations in disease severity, suggesting that individuals with milder form of diabetes tend to experience higher quality of life [34,37–39]. Additionally, previous research using the EQ-5D index has shown that the average EQ-5D-5L index value ranges from 0.77 to 0.95 for patients with diabetes [34,40–42]. In our study, the average EQ-5D-5L score was 0.92, which is within the range reported in a previous local study [17]. Finally, the diabetes P4P program in Taiwan was associated with better QoL among all patients and those with gastrointestinal comorbidities. The results were robust because we controlled for the confounders above, and PSW was applied to prevent selection bias.

The probable reasons for the P4P program having spillover effects on a better EQ-5D index among patients with gastrointestinal comorbidities. The diabetes P4P program in Taiwan not only has an incentive mechanism for adherence to physician guidelines but also integrates a shared care network (SCN) [21] that has distinctive characteristics, such as continuing education, coordinated care and regular follow-up, thereby improving clinical outcomes in diabetic patients with gastrointestinal comorbidities. First, in Taiwan, participation in the diabetes P4P program requires that health care professionals undergo regular continuing education. Although hepatitis HCV-specific training has become more prominent in recent years, particularly after the Ministry of Health and Welfare (MoHW) introduced the National Hepatitis C Policy Guideline (2018–2025) [43], it is likely that even before 2017, general education on managing comorbidities may have increased health care professionals’ awareness of liver-related complications in patients with diabetes. Second, the P4P program was developed on the basis of the principles of the chronic care model (CCM) [21], in which one of the core components is effective coordination among medical specialties. In addition to common interdisciplinary collaborations such as those with ophthalmology or cardiology [44], some health care institutions have initiated integrative care pathways between endocrinology and gastroenterology for patients with diabetes [45]. Systematic reviews have shown that such integrative care models can significantly enhance patients’ quality of life [46]. Third, more health care institutions may be unable to implement integrated care with gastroenterology departments due to various institutional constraints [47]. However, patients enrolled in the P4P program typically attend regular follow-up visits [48], which enables physicians involved in diabetes management—such as endocrinologists or family medicine specialists—to become more aware of gastrointestinal issues. Consequently, these patients are more likely to be proactively referred to gastroenterology for further evaluation [2,49]. Alternatively, patients may be referred by diabetes care physicians to health educators for counseling on lifestyle modifications, such as interventions addressing alcohol cessation (i.e., alcohol consumption leading to severe hepatitis [6,35]), which is associated with better health-related QoL among patients with diabetes [50].

There were several limitations in our study. First, we could not control for all of the factors related to QoL scores; however, we controlled for possible and important socioeconomic and clinical factors and applied those factors in the PSW analysis to prevent selection bias. Second, there were some concerns about the low rate of reports of gastrointestinal conditions. To address this problem, we asked the interviewers to pay attention to the results reported by the patients. However, we cannot completely rule out the possibility of underreporting gastrointestinal conditions. In our study, the prevalence of gastrointestinal conditions was 19% (284/1477), which was slightly higher than that reported in other studies conducted in Asia if we considered only HBV infection in individuals with type 2 diabetes mellitus [51]. We tried to use the NHIA’s national claims data to verify the results, and we first reported that in 2013, the average age of patients with diabetes from the national database was 62.51 years (data not shown), which is within the range of our sample. The sex distribution was also identical (data not shown). Additionally, the prevalence rate of gastrointestinal conditions from the CIC index from the national database was approximately 24% (data not shown), which indicates that the lower prevalence rate (19%) of gastrointestinal conditions in our study was not severe, thus supporting the findings that the P4P program has more benefits for patients with gastrointestinal comorbidities. In the future, a link to the database of the NHIA should be established to verify patients with gastrointestinal comorbidities. Third, QoL was assessed in a cross-sectional manner, thus decreasing the generalizability of the results. In the future, patients should be followed up to evaluate their subsequent quality of life, and a quasi-experimental design incorporating preceding QoL outcomes should be used. Fourth, we cannot rule out the possibility that patients referred by physicians or health care personnel to conduct questionnaires may have certain characteristics that incur bias. However, after adjustment through PSW, the P4P and non-P4P groups categorized from all patients were comparable. Therefore, the bias introduced by referrals can be mitigated. Fifth, our data cover the year 2016, and although this may raise concerns about data currency, we believe the findings remain relevant. The definitions and implementation of the diabetes P4P program may have evolved since its initiation in 2001, which could affect the interpretation of our results. Prior to 2012, the diabetes P4P proposal underwent nine revisions, characterized by significant reforms such as the 1) inclusion of outcome-based incentives, 2) the implementation of pay-for-improvement mechanisms, and 3) initially, introduced as a pilot program, the diabetes P4P was formally integrated into the official payment system. From 2012 to the end of our data period in 2016, as well as after 2016, there were no significant changes in the diabetes P4P program. In recent years, the NHIA has merged with the early chronic kidney disease (CKD) P4P [52]. Consequently, this new initiative may benefit diabetic patients with CKD complications but potentially has a limited effect on gastrointestinal conditions. In summary, although the data in this study may be old, the conclusions remain potentially applicable. Sixth, this study employs a two-part model. Under the conditions of α = 0.05, power = 0.8, and an effect size of 0.1 (considering both the binomial and normal parts), the minimum sample size required is 96 [53]. In this study, there were 249 diabetic patients with gastrointestinal conditions. Therefore, the sample size in this study may be sufficient. However, since the actual sample size is not large, caution should be exercised when interpreting the results of this study.

## Conclusions

Our findings suggest that participation in the diabetes P4P program is associated with improved health-related quality of life (QoL) among all patients with type 2 diabetes, including those with gastrointestinal comorbidities. A P4P model incorporating continuing education, coordinated care, and regular follow-up may be particularly beneficial in enhancing QoL for patients with gastrointestinal complications.

## Supporting information

S1 Table Standardized mean difference and percent reduction before/after propensity score weighting for every variable and its categories. (DOCX)
