# Supplementary material for: Is the pay-for-performance program associated with better quality of life among type 2 diabetes patients, including those with gastrointestinal conditions, in Taiwan? A cross-sectional survey
Source: PLoS One. 2025 Aug 22;20(8):e0328910. doi: 10.1371/journal.pone.0328910 (PMC12373241; doi:10.1371/journal.pone.0328910)
Supplement: S1 Table — (DOCX) [file pone.0328910.s001.docx]

**Appendix**

After applying PSW, the absolute values of the standardized mean differences (SMDs) between the P4P and non-P4P groups significantly decreased for every variable and its related categories. The largest of these differences was 0.022 (absolute value), which is much less than the upper limit of 0.25 recommended by another study[1, 2].

S1 Table Standardized mean difference and percent reduction before/after propensity score weighting for every variable and its categories

| **Variable** | **Before/After PSW** | **Standardized mean difference** | **Percent reduction** |
| --- | --- | --- | --- |
| **Overall** | Before | 0.567 |  |
|  | After | -0.021 | 96 |
| **Age** | Before | 0.289 |  |
|  | After | -0.019 | 93 |
| **Male** | Before | -0.091 |  |
|  | After | 0.021 | 77 |
| **Income** |  |  |  |
| < U.S.$1,000 | Before | -0.077 |  |
|  | After | 0.023 | 70 |
| U.S.$1,000-$2,000 | Before | 0.059 |  |
|  | After | -0.008 | 85 |
| U.S.$2,001-$3,333 | Before | 0.046 |  |
|  | After | -0.022 | 53 |
| >U.S.$3,333 | Before | -0.005 |  |
|  | After | -0.001 | 0 |
| **Employment** |  |  |  |
| Full-time without a shift | Before | 0.037 |  |
|  | After | -0.012 | 67 |
| Full-time with a shift | Before | 0.102 |  |
|  | After | -0.012 | 88 |
| Retired | Before | -0.163 |  |
|  | After | 0.0189 | 88 |
| Part-time | Before | 0.0606 |  |
|  | After | -0.006 | 89 |
| Self-employed | Before | 0.005 |  |
|  | After | 0.005 | 0 |
| Others | Before | 0.069 |  |
|  | After | -0.005 | 93 |
| **Marital status** |  |  |  |
| Married | Before | -0.128 |  |
|  | After | 0.008 | 94 |
| Unmarried | Before | 0.132 |  |
|  | After | -0.003 | 97 |
| Widowed | Before | 0.004 |  |
|  | After | -0.009 | 0 |
| Other | Before | 0.069 |  |
|  | After | 0.002 | 97 |
| **Education** |  |  |  |
| Below elementary school | Before | -0.052 |  |
|  | After | 0.005 | 90 |
| Junior high school | Before | -0.054 |  |
|  | After | -0.011 | 79 |
| Junior college | Before | 0.083 |  |
|  | After | 0.005 | 94 |
| Senior high school | Before | -0.013 |  |
|  | After | 0.012 | 11 |
| University | Before | 0.036 |  |
|  | After | -0.014 | 62 |
| Graduate or doctoral degree | Before | 0.115 |  |
|  | After | -0.002 | 98 |
| **Level of urbanization** |  |  |  |
| High-level | Before | -0.315 |  |
|  | After | 0.003 | 98 |
| Median-level | Before | 0.250 |  |
|  | After | -0.010 | 96 |
| Emerging | Before | 0.104 |  |
|  | After | 0.008 | 92 |
| Common | Before | 0.103 |  |
|  | After | 0 | 100 |
| **Treatment** |  |  |  |
| Exercise and diet control | Before | 0.2661 |  |
|  | After | -0.007 | 97 |
| Oral medications only | Before | -0.233 |  |
|  | After | -0.006 | 97 |
| Oral medications and use of insulin | Before | 0.096 |  |
|  | After | 0.010 | 89 |
| Use of insulin only | Before | 0.036 |  |
|  | After | 0.003 | 92 |
| **Multiple shots of insulin per day** | Before | 0.008 |  |
|  | After | -0.002 | 77 |
| **Gastrointestinal conditions** | Before | 0.019 |  |
|  | After | -0.007 | 64 |
| **No complications** | Before | 0.141 |  |
|  | After | -0.002 | 98 |
| **Previous hospital admission within one year** | Before | 0.008 |  |
|  | After | -0.002 | 77 |

**References**

1. Rubin, D. B. (2001). “Using Propensity Scores to Help Design Observational Studies: Application to the Tobacco Litigation.” Health Services and Outcomes Research Methodology 2:169–188.

2. Stuart EA. Matching Methods for Causal Inference: A Review and a Look Forward. Stat Sci. 2010;25(1):1-21. <https://doi.org/10.1214/09-Sts313>. PMID: WOS:000281096800001.
